# The clinical evaluation of atlas-based auto-segmentation for automatic contouring during cervical cancer radiotherapy

**DOI:** 10.3389/fonc.2022.945053

**Published:** 2022-08-02

**Authors:** Yi Li, Wenjing Wu, Yuchen Sun, Dequan Yu, Yuemei Zhang, Long Wang, Yao Wang, Xiaozhi Zhang, Yongkai Lu

**Affiliations:** ^1^ Department of Radiation Oncology, The First Affiliated Hospital of Xi’an Jiaotong University, Xi’an, China; ^2^ Department of Radiological Health, Xi’an Center for Disease Control and Prevention, Xi’an, China; ^3^ Department of Radiation Oncology, Tangdu Hospital, the Second Affiliated Hospital of Air Force Medical University, Xi’an, China

**Keywords:** atlas, auto-segmentation, automatic contouring, cervical cancer, radiotherapy

## Abstract

**Purpose:**

Our purpose was to investigate the influence of atlas library size and CT cross-slice number on the accuracy and efficiency of the atlas-based auto-segmentation (ABAS) method for the automatic contouring of clinical treatment volume (CTV) and organs at risk (OARs) during cervical cancer radiotherapy.

**Methods:**

Of 140 cervical cancer patients, contours from 20, 40, 60, 80, 100, and 120 patients were selected incrementally to create six atlas library groups in ABAS. Another 20 tested patients were automatically contoured with the ABAS method and manually contoured by the same professional oncologist. Contours included CTV, bladder, rectum, femoral head-L, femoral head-R, and spinal cord. The CT cross-slice numbers of the 20 tested patients included 61, 65, 72, 75, 81, and 84. The index of dice similarity coefficients (DSCs) and Hausdorff distance (HD) were used to assess the consistency between ABAS automatic contouring and manual contouring. The randomized block analysis of variance and paired t-test were used for statistical analysis.

**Results:**

The mean DSC values of “CTV, bladder, femoral head, and spinal cord” were all larger than 0.8. The femoral head and spinal cord showed a high degree of agreement between ABAS automatic contouring and manual contouring, with a mean DC >0.80 and HD <1 cm in all atlas library groups. A *post-hoc* least significant difference comparison indicated that no significant difference had been found between different atlas library sizes with DSC and HD values. For ABAS efficiency, the atlas library size had no effect on the time of ABAS automatic contouring. The time of automatic contouring increased slightly with the increase in CT cross-slice numbers, which were 99.9, 106.8, 114.0, 120.6, 127.9, and 134.8 s with CT cross-slices of 61, 65, 72, 75, 81, and 84, respectively.

**Conclusion:**

A total of 20 atlas library sizes and a minimum CT cross-slice number including CTV and OARs are enough for ensuring the accuracy and efficiency of ABAS automatic contouring during cervical cancer radiotherapy.

## Introduction

Radiotherapy, as an independent treatment strategy, plays an important role in cervical cancer treatment (1). Delineating the clinical target volume (CTV) and organs at risk (OARs) precisely is essential to ensuring the curative effect of radiotherapy. However, manual contouring is a time-consuming and complex process. Significant variance can be detected among contours that were delineated by different radiation oncologists or the same oncologist at different times (2–4). Compared with the manual segmentation process, atlas-based auto-segmentation (ABAS) could not only significantly save time but also reduce subjective bias among different radiation oncologists. In ABAS, segmented structures from the atlas library are propagated onto a subject image using the deformable image registration algorithm. Because multiple-ABAS uses a voting scheme for determining whether a voxel is inside or outside the structure, it is more susceptible to topological artifacts compared with single-ABAS (5).

The atlas library should be set before applying the ABAS method for delineating contours automatically. According to published results, multiple-ABAS could overcome the issues encountered with single-ABAS, such as large discrepancies in volume and location between the atlas library and subject data (6). However, the atlas library size varied a lot in different cases and no authorized guideline could be taken as the reference (7–10). Although there are several reports about atlas library size in pelvic radiotherapy (11–13), especially for endometrial and cervical cancer (14), limited atlas library groups and fewer test cases were available on the impact of atlas library size on the accuracy and efficiency of ABAS, which may lead to an inaccurate conclusion. Rare works have researched the effect of CT cross-slice numbers on the ABAS-performed efficiency. Therefore, the aim of our study is to investigate the influence of the atlas library size and CT cross-slice number on the accuracy and efficiency of ABAS automatic contouring and establish an optimal strategy for ABAS with atlas library size and CT cross-slice number during cervical radiotherapy.

## Material and methods

### Patients

This retrospective study included 140 patients with newly diagnosed, pathologically confirmed stage II–III cervical cancer (7th edition of the AJCC staging system). All patients were treated with volume-modulated arc therapy (VMAT) technology in the Radiation Oncology Department at the First Affiliated Hospital of Xi’an Jiaotong University from October 2020 to October 2021. The VMAT plan was delivered at the prescribed dose of 50 Gy (25 fractions) to the cervical tumor. Neoadjuvant, concurrent, or adjuvant chemotherapy was recommended for patients.

### CT simulation

A total of 140 planning CT images from 140 patients were collected. All patients were immobilized using thermoplastic body mold in the supine position with hands raised and arms crossed with elbows on the top of the head during CT simulation. All patients were instructed that the rectum should be completely empty and the bladder should be filled with 300 ml of water 2 h before CT scanning. Each patient received a helical CT scanning under free breathing conditions on a 16-slice CT scanner (Big Bore, Philips Medical Systems, Cleveland, OH). The scanning parameters were as follows: pixel spacing 1.1543 mm × 1.1543 mm, matrix 512 × 512, pitch 0.85, 120 kV, 400 mAs, thickness 3 mm, and layer spacing 3 mm. The scanned images were sent to a MIM Maestro version 6.7 software (Cleveland, OH, USA) with ABAS function based on a computer (Intel(R), Xeon(R), E5-1603 CPU, 2.8 GHz, four processors, 32 G RAM).

### Manual contouring

The professional oncologist manually contoured CTV and OARs on the CT-scanned images of all patients with reference to RTOG 0418 (15) and consensus guidelines (16, 17). CTV included the tumor volume and whole pelvic nodal volume. OARs included the bladder, femoral head-L, femoral head-R, rectum, and spinal cord. CTV and OARs were delineated manually by the same professional oncologist with the mediastinum window setting (L = 40 Hu, W = 350 Hu) to make the interobserver lowest as soon as possible, which were used as the atlas library data and tested the segmentation ground truth.

### Atlas library creation and automatic contouring

Of 140 cervical cancer patients, 120 patients were randomly registered in six different atlas library groups with 20, 40, 60, 80, 100, and 120 patients incrementally for ABAS. The automatic segmentation process was performed in the 20 remaining tested patients with ABAS. The details are shown in **Table 1**. During library construction, a template subject was assigned, and the remaining subjects were registered to the template subject separately. To minimize the bias and maintain the consistency of the registration alignment, an additional intervention during registration was prohibited. The ABAS algorithm automatically matched the atlas subject according to the input tested set. The optimal number of the matched atlas template was set to three during ABAS automatic contouring. Based on the intensity and the freeform cubic spline interpolation, contours of CTV and OARs were deformed, registered, and transferred to the test set. The CT cross-slice number of the selected automatically contoured patients ranged from 61 to 84 (the average number was 72 slices per patient). Then, manual correction of ABAS automatic contours was performed in the 20 tested patients.

**Table 1:** Characteristics of atlas library and tested patients.

	TestN = 20	Size of atlas library					
		N = 20	N = 40	N = 60	N = 80	N = 100	N = 120
Mean age (SD)	50.8 (11.3)	52.1 (12.5)	53.4 (12.2)	53.8 (11.7)	52.8 (12.3)	51.8 (11.9)	54.8 (12.6)
Mean height (SD), cm	157.0 (7.4)	158.0 (6.9)	159.7 (6.4)	157.4 (7.6)	158.4 (6.9)	157.4 (7.8)	159.4 (7.5)
Mean weight (SD), kg	62.7 (8.5)	62.5 (7.1)	61.3 (6.8)	61.7 (7.5)	62.9 (7.6)	61.6 (8.3)	62.7 (7.3)
Mean BMI (SD), kg/m^2^	25.4 (4.5)	25.0 (4.3)	24.0 (3.9)	24.9 (4.1)	25.1 (3.8)	24.9 (4.2)	24.7 (4.4)

### Quantitative evaluation of accuracy and efficiency of ABAS automatic contouring

Contours generated by ABAS automatic contours were compared with manually corrected ABAS automatic contours. The Dice similarity coefficient (DSC) and Hausdorff distance (HD) were used to evaluate the accuracy of automatic contouring (14). DSC was defined as DSC = 2|A∩B|/(A+B) with A equaling the volume of automatic contouring volume and B equaling the manual contouring volume. The results of DSC were between 0 and 1, where 0 represents no intersection and 1 reflects a perfect overlap of structures. In contrast, HD considered the degree of mismatch between two surfaces based on contour boundaries, eliminating the ambiguity of the volume-based DSC metric. Moreover, we timed the whole process to evaluate the efficiency of the ABAS method.

### Statistical method

Random analysis of variance and paired-sample t-test were used to analyze the accuracy of automatic contouring results between the different atlas library sizes and CT cross-slice numbers. P < 0.05 was regarded as statistically significant. SPSS 22.0 was used for statistics analysis.

## Results

### DSC and HD values of contours of “CTV and OARs”

**Figure 1:**
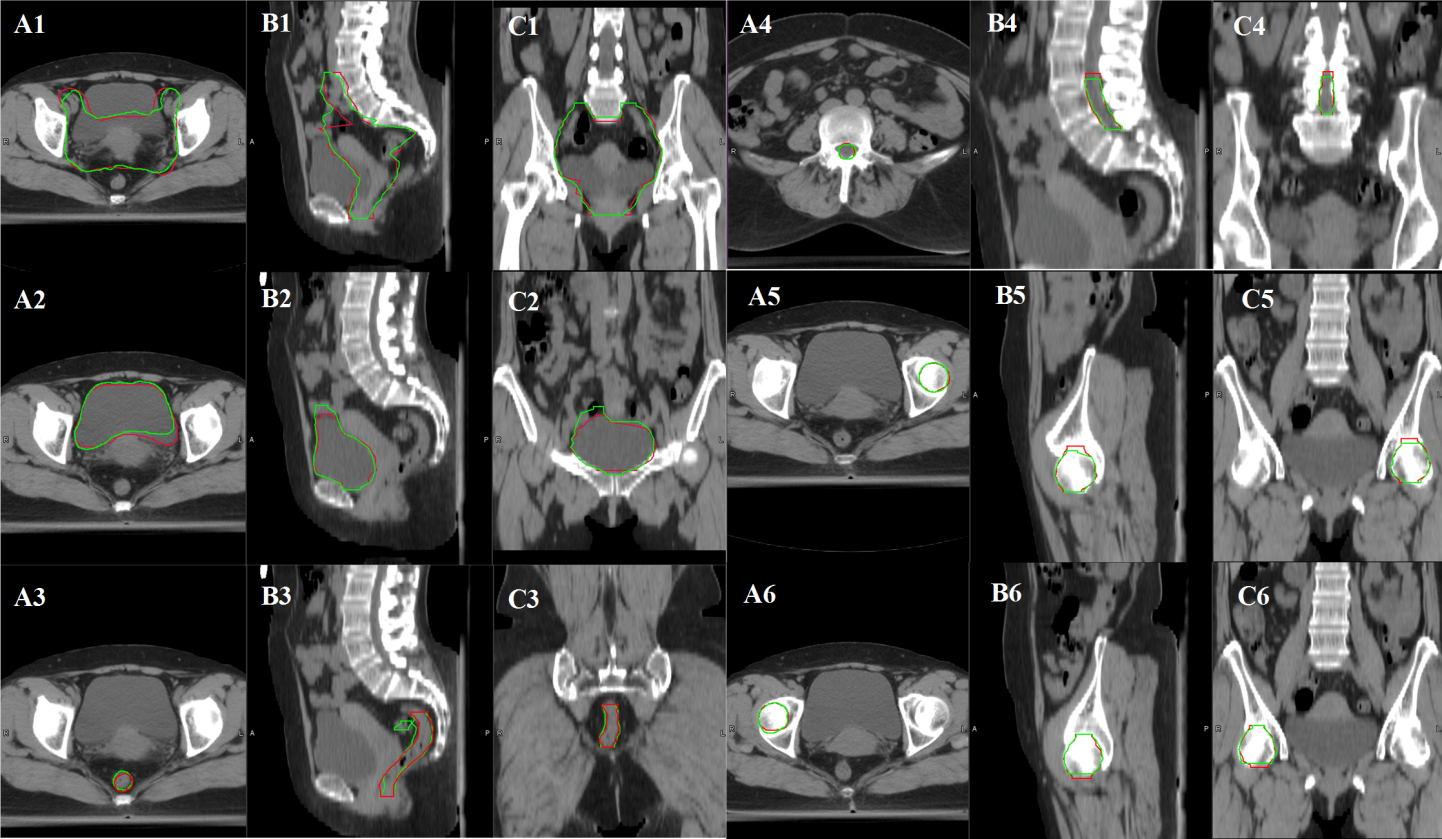
Variance between manual contours and ABAS automatic contours in CTV and OARs of cervical cancer patients with an atlas library size of 20 (**A1, B1, C1**: CTV contour comparison, **A2, B2, C2**: bladder contour comparison, **A3, B3, C3**: rectum contour comparison, **A4, B4, C4**: spinal cord comparison, **A5, B5, C5**: femoral head-L contour comparison, **A6, B6, C6**: femoral head-R contour comparison. Manual contours in red color, and automatic contours in green color).

A total of 20 cervical cancer patients’ CTV and OARs were delineated both by a radiation oncologist manually and ABAS automatically with an atlas library size of 20. The consistency between automatic and manual segmentation was assessed with DSC and HD values. According to the results, the mean DSC values of “CTV, bladder, femoral head, and spinal cord” were all larger than 0.8. However, the mean DSC value and HD value of the rectum were 0.695 and 2.508 cm, respectively. Therefore, the contours of the rectum needed to be corrected greatly before clinical application as shown in **Figure 1** and **Table 2**.

**Table 2:** DSC and HD value of CTV and OARs with an atlas library size of 20.

Structure	DSC	HD (cm)
CTV	0.816 ± 0.046	2.195 ± 0.340
Bladder	0.866 ± 0.035	1.591 ± 0.340
Rectum	0.685 ± 0.048	2.508 ± 0.559
Femoral head-L	0.867 ± 0.039	0.843 ± 0.369
Femoral head-R	0.873 ± 0.047	0.789 ± 0.260
Spinal cord	0.858 ± 0.052	0.604 ± 0.205

### The influence of atlas library size on the accuracy of ABAS automatic segmentation

ABAS was used to delineate the contours of “CTV and OARs” of cervical cancer patients under different atlas library groups (20, 40, 60, 80, 100, and 120, respectively). The DSC and HD values of CTV and OARs in the different atlas library groups are shown in **Table 3** and **Table 4**. The results showed that different atlas library sizes had little impact on the accuracy of automatic contouring. Randomized block analysis of variance was adopted to further investigate the influence of atlas library size on the automatic contouring accuracy. *Post-hoc* least significant difference (LSD) comparisons indicated that no significant difference was found between different atlas library groups as shown in **Table 5** and **Table 6**. Therefore, an atlas library size of 20 was enough to delineate CTV and OARs automatically with the ABAS method during cervical patients’ radiotherapy.

**Table 3:** The DSC value of CTV and OARs in different atlas library groups (mean ± SD).

	20	40	60	80	100	120
CTV	0.816 ± 0.046	0.818 ± 0.046	0.819 ± 0.049	0.814 ± 0.036	0.819 ± 0.061	0.810 ± 0.046
Bladder	0.866 ± 0.035	0.857 ± 0.043	0.860 ± 0.034	0.854 ± 0.047	0.855 ± 0.043	0.861 ± 0.046
Rectum	0.685 ± 0.048	0.682 ± 0.041	0.693 ± 0.042	0.689 ± 0.048	0.682 ± 0.048	0.692 ± 0.053
Femoral head-L	0.867 ± 0.039	0.868 ± 0.047	0.865 ± 0.037	0.875 ± 0.046	0.864 ± 0.046	0.870 ± 0.038
Femoral head-R	0.873 ± 0.047	0.865 ± 0.041	0.870 ± 0.045	0.867 ± 0.042	0.869 ± 0.046	0.879 ± 0.037
Spinal cord	0.858 ± 0.052	0.857 ± 0.058	0.856 ± 0.053	0.851 ± 0.038	0.859 ± 0.037	0.858 ± 0.051

**Table 4:** The HD value of CTV and OARs in different atlas library groups (mean ± SD, cm).

	20	40	60	80	100	120
CTV	2.195 ± 0.340	2.335 ± 0.223	2.310 ± 0.265	2.345 ± 0.255	2.363 ± 0.275	2.074 ± 0.308
Bladder	1.591 ± 0.340	1.612 ± 0.257	1.566 ± 0.332	1.571 ± 0.293	1.614 ± 0.272	1.559 ± 0.298
Rectum	2.045 ± 0.562	2.097 ± 0.554	1.967 ± 0.381	1.956 ± 0.401	2.039 ± 0.429	1.925 ± 0.449
Femoral head-L	0.843 ± 0.369	0.727 ± 0.230	0.774 ± 0.204	0.690 ± 0.217	0.725 ± 0.297	0.730 ± 0.228
Femoral head-R	0.789 ± 0.260	0.730 ± 0.235	0.700 ± 0.140	0.635 ± 0.185	0.698 ± 0.195	0.673 ± 0.167
Spinal cord	0.604 ± 0.205	0.767 ± 0.336	0.640 ± 0.225	0.626 ± 0.267	0.677 ± 0.213	0.636 ± 0.161

**Table 5:** Results of *post-hoc* least significant difference (LSD) with DSC value among different atlas library groups.

Atlasgroup	Atlasgroup	CTV	Bladder	Rectum	Femoral head-L	Femoral head-R	Spinal cord
20	40	0.753	0.694	0.291	0.592	0.610	0.240
	60	0.519	0.094	0.490	0.237	0.492	0.191
	80	0.954	0.372	0.268	0.883	0.877	0.062
	100	0.508	0.260	0.644	0.320	0.705	0.199
	120	0.745	0.886	0.104	0.592	0.722	0.163
40	20	0.753	0.694	0.291	0.592	0.610	0.240
	60	0.740	0.040	0.712	0.514	0.859	0.892
	80	0.797	0.200	0.958	0.697	0.722	0.477
	100	0.727	0.131	0.551	0.644	0.895	0.910
	120	0.991	0.591	0.558	1.000	0.877	0.820
60	20	0.519	0.094	0.490	0.237	0.492	0.191
	40	0.740	0.040	0.712	0.514	0.859	0.892
	80	0.556	0.425	0.673	0.299	0.594	0.565
	100	0.986	0.573	0.819	0.848	0.757	0.982
	120	0.749	0.124	0.342	0.514	0.740	0.928
80	40	0.954	0.372	0.268	0.883	0.877	0.062
	60	0.797	0.200	0.958	0.697	0.722	0.477
	80	0.556	0.425	0.673	0.299	0.594	0.565
	100	0.545	0.813	0.516	0.395	0.823	0.550
	120	0.788	0.453	0.594	0.697	0.841	0.628
100	20	0.508	0.260	0.644	0.320	0.705	0.199
	40	0.727	0.131	0.551	0.644	0.895	0.910
	60	0.986	0.573	0.819	0.848	0.757	0.982
	80	0.545	0.813	0.516	0.395	0.823	0.550
	120	0.736	0.325	0.240	0.644	0.981	0.910
120	20	0.745	0.886	0.104	0.592	0.722	0.163
	40	0.991	0.591	0.558	1.000	0.877	0.820
	60	0.749	0.124	0.342	0.514	0.740	0.928
	80	0.788	0.453	0.594	0.697	0.841	0.628
	100	0.736	0.325	0.240	0.644	0.981	0.910

**Table 6:** Results of *post-hoc* least significant difference (LSD) with HD value among different atlas library groups.

Atlasgroup	Atlasgroup	CTV	Bladder	Rectum	Femoral head-L	Femoralhead-R	Spinal cord
20	40	0.195	0.829	0.723	0.169	0.356	0.064
	60	0.287	0.789	0.602	0.413	0.164	0.637
	80	0.164	0.834	0.551	0.069	0.067	0.773
	100	0.120	0.813	0.970	0.162	0.153	0.340
	120	0.261	0.737	0.421	0.180	0.071	0.675
40	20	0.195	0.829	0.723	0.169	0.356	0.064
	60	0.816	0.629	0.382	0.574	0.638	0.098
	80	0.922	0.670	0.343	0.654	0.138	0.067
	100	0.791	0.983	0.696	0.981	0.610	0.240
	120	0.117	0.581	0.248	0.971	0.372	0.088
60	20	0.287	0.789	0.602	0.413	0.164	0.637
	40	0.816	0.629	0.382	0.574	0.638	0.098
	80	0.741	0.954	0.941	0.313	0.309	0.854
	100	0.618	0.614	0.628	0.558	0.969	0.628
	120	0.060	0.946	0.777	0.599	0.672	0.958
80	40	0.164	0.834	0.551	0.069	0.067	0.773
	60	0.922	0.670	0.343	0.654	0.138	0.067
	80	0.741	0.954	0.941	0.313	0.309	0.854
	100	0.867	0.655	0.576	0.671	0.328	0.504
	120	0.113	0.900	0.834	0.628	0.551	0.896
100	20	0.120	0.813	0.970	0.162	0.153	0.340
	40	0.791	0.983	0.696	0.981	0.610	0.240
	60	0.618	0.614	0.628	0.558	0.969	0.628
	80	0.867	0.655	0.576	0.671	0.328	0.504
	120	0.108	0.567	0.443	0.952	0.701	0.591
120	20	0.261	0.737	0.421	0.180	0.071	0.675
	40	0.117	0.581	0.248	0.971	0.372	0.088
	60	0.060	0.946	0.777	0.599	0.672	0.958
	80	0.113	0.900	0.834	0.628	0.551	0.896
	100	0.108	0.567	0.443	0.952	0.701	0.591

### Influence of atlas library size and CT cross-slice number on the efficiency of ABAS automatic contouring

One-way ANOVA was adopted to investigate the influence of atlas library size and CT cross-slice number on the efficiency of ABAS automatic contouring. The atlas library size had no effect on the time of ABAS automatic contouring, as shown in **Table 7**. The times of ABAS automatic contouring increased slightly with the increase in CT cross-slice numbers, which were 99.9, 106.8, 114.0, 120.6, 127.9, and 134.8 s with CT cross-slice numbers of 61, 65, 72, 75, 81, and 84, respectively, as shown in **Table 8**. As a result, a minimum CT cross-slice number was suggested for delineating the CTV and OARs automatically during cervical cancer radiotherapy.

**Table 7:** The time of ABAS automatic segmentation among different atlas library sizes.

	Time (s)	Range	*P* value
20	114.3	108.7~119.9	
40	115.2	109.2~121.1	
60	114.1	100.2~128.1	0.974
80	115.8	103.9~127.8	
100	117.2	104.2~130.1	
120	119.2	105.8~136.7	

**Table 8:** Time for ABAS automatic contouring among different CT cross-slice numbers.

CT cross-slice number	61	65	72	75	81	84	P
Time (s)	99.9	106.8	114.0	120.6	127.9	134.8	0.032

## Discussion

Our study investigated the influence of atlas library size and patients’ CT cross-slice number on the accuracy and efficiency of ABAS and established an optimal atlas library and CT cross-slice for automatic contouring during cervical cancer radiotherapy, which was rarely discussed in previous studies. ABAS was introduced to delineate CTV and OARs automatically and reduce interobserver and intra-observer contouring variability. Previous studies (3, 18) demonstrated that contouring time could be reduced greatly using the ABAS method for head and neck cancer. Voet et al. (19) demonstrated that the delineation time was reduced from 180 to 66 min using the ABAS method despite necessary auto-contour editing. However, it should be noted that the manually corrected contouring times were difficult to assess accurately because manual correction times varied significantly with different oncologists or the same oncologist at different times. Therefore, we mainly focused on ABAS automatic contouring time without considering the manually corrected time. In clinical practice, it was a challenge to select the appropriate atlas library size. In our study, we found that a large atlas library size was not necessary for ABAS automatic contouring and an atlas library size of 20 could be enough to insure the accuracy of automatic contouring with the ABAS method. However, some authors validated ABAS based on a higher number of subjects for male pelvis CT images compared to what is concluded in the present article (20, 21). Some authors validated ABAS based on a lower number of subjects for prostate cancers if compared to what is concluded in the present article (7, 11). In addition, Ducote et al. concluded that the performance of ABAS was relatively insensitive to atlas size for various head and neck cancers (22). Kim et al. demonstrate that a different atlas library size had no impact on the accuracy of ABAS-OAR automatic contouring and the segmentation accuracy of ABAS-CTV improved with increasing library size with ABAS (14). In our opinion, the size of the atlas library is not an independent factor in determining the quality of auto-segmentation.

DSC was used to assess the overlap ratio between ABAS automatic contouring and manual contouring. In our study, we found that ABAS showed acceptable accuracy in delineating CTV and parts of OARs such as bladder, femoral head-L, femoral head-R, and spinal cord, with the mean DSC of 0.816, 0.866, 0.867, 0.873, and 0.858, which was larger than a good overlap standard of 0.7 according to a published paper (8). The femoral head and spinal cord showed a high degree of agreement between ABAS automatic contouring and manual contouring, with a mean DC >0.80 and HD <1 cm in all atlas library groups, which was similar to the result by Kim et al. (14). For ABAS efficiency, we found that the atlas library size had no effect on the time of ABAS automatic contouring and automatic contouring time increased with the increase in CT cross-slice number. Therefore, we suggested a minimum CT cross-slice number, which could include CTV and OARs, for ABAS automatic contouring during cervical patient radiotherapy.

Earlier work by Stuart Greenham et al. (12) evaluated the performance of ABAS automatic contouring, and the results showed that the bladder and CTV, which are the two largest structures in the pelvis, were delineated precisely in delineating the pelvic anatomy, which was similar with the results of our study. Among all the OARs, the bladder, femoral heads, and spinal cord achieved a higher DSC value and a lower HD value. This may be due to the relatively clear boundary and high contrast between these structures and the background. However, it should be noted that ABAS could not precisely delineate the rectum in this study. There are two reasons for this result. First, this could be caused by the unclear boundaries and the massive diversity in sizes, shapes, and locations for different patients. Even an experienced radiotherapy oncologist sometimes had to delineate the rectum boundary by experience instead of by images. To get accurate delineating results, manual corrections were suggested for rectum automatic contouring. In accordance with radiotherapy instructions, the rectum should be completely empty before CT scanning and the CT scanning time should be enough to acquire the images with contrast-enhanced ultrasound properly, which could improve the clarity of rectum boundaries and contouring accuracy. However, it was hard to control in clinical practice, which could result in poor automatic contouring and inaccurate manual contouring. Second, the atlas-based auto-segmentation method lacks necessary intervention methodologies and provides little final control by the medical doctor over the segmentation. Recently, auto-segmentation methodology with a user-defined template for the library construction could handle the variations in rectum anatomy. Luddemann et al. (23) have evaluated an interactive graph-based segmentation algorithm with a user-defined template by comparing the computer-assisted segmentation results with manual expert segmentation of the rectum/sigmoid colon and yielded a DSC of 83.58 ± 4.08% in gynecological brachytherapy. Therefore, auto-segmentation with a user-defined template can be used for rectosigmoid colon segmentation in gynecological external-beam radiation and gynecological brachytherapy.

Recently, the scope of auto-segmentation has been expanded to arterial intelligence (AI)-based contouring using deep learning algorithms (24–29). Earlier work by Liu (30) demonstrated that the mean DSC values of deep learning-based methods were 0.924 for the bladder, 0.906 for the femoral head-L, 0.900 for the femoral head-R, 0.791 for the rectum, and 0.827 for the spinal cord. The results of ABAS in this study had comparable performance with deep learning methods in the spinal cord but an inferior performance in the bladder, rectum, femoral head-L, and femoral head-R. The deep learning-based method outperformed the ABAS method in OAR automatic contouring. However, many radiotherapy departments have to use ABAS to automatically contour the target area and normal organs due to a lack of deep learning equipment or condition. This study provided a method for selecting appropriate atlas library sizes and CT cross-slices for the departments with ABAS.

There are several limitations in the current study. First, a cohort of patients was included in all the atlases used in six different atlas libraries. This pool could be overrepresented, and this issue could influence somehow the results of the work. Therefore, further investigations including a large number of independently tested patients are needed for evaluating the efficacy of the current ABAS library. Second, the research of this approach only evaluated the influence of CT cross-slice numbers on the time of ABAS automatic contouring. Future work will be required to evaluate the influence of CT cross-slice number on DSC and HD values of ABAS automatic contouring.

## Conclusion

In our study, 20 atlases and a minimum CT cross-slice number could insure the accuracy and efficiency of ABAS automatic contouring during cervical cancer radiotherapy. Considering that ABAS could not delineate the rectum organ accurately, manual correction by a radiation oncologist is necessary. The data that we used in this study was from only one department, which means that the model may not apply to data with different situations. A larger dataset and more data sources could make the result more robust and have a better generalization capability.

## Data availability statement

The original contributions presented in the study are included in the article/supplementary material. Further inquiries can be directed to the corresponding author/s.

## Author contributions

WW and XZ planned and designed the research. YL, YS, and DY performed the experiment and wrote the manuscript. WW, YKL, and YZ revised the manuscript. YKL, LW, and YW analyzed the data. All authors contributed to the article and approved the submitted version.

## Funding

This work was supported by the Key Research and Development Projects of Shaanxi Province, China (No. 2022SF-440).

## Acknowledgments

All authors in this study and many colleagues helped to collect and analyze data. Their support and helps should be appreciated.

## Conflict of interest

The authors declare that the research was conducted in the absence of any commercial or financial relationships that could be construed as a potential conflict of interest.

## Publisher’s note

All claims expressed in this article are solely those of the authors and do not necessarily represent those of their affiliated organizations, or those of the publisher, the editors and the reviewers. Any product that may be evaluated in this article, or claim that may be made by its manufacturer, is not guaranteed or endorsed by the publisher.

## References

[1] Kokka F Bryant A Brockbank E Powell M Oram D. Hysterectomy with radiotherapy or chemotherapy or both for women with locally advanced cervical cancer. Cochrane Database Syst Rev (2015) 4:D10260. doi: 10.1002/14651858.CD010260.pub2 25847525

[2] Young AV Wortham A Wernick I Evans A Ennis RD. Atlas-based segmentation improves consistency and decreases time required for contouring postoperative endometrial cancer nodal volumes. Int J Radiat Oncol Biol Phys (2011) 79:943–7. doi: 10.1016/j.ijrobp.2010.04.063 21281897

[3] Teguh DN Levendag PC Voet PW Al-Mamgani A Han X Wolf TK. Clinical validation of atlas-based auto-segmentation of multiple target volumes and normal tissue (swallowing/mastication) structures in the head and neck. Int J Radiat Oncol Biol Phys (2011) 81:950–7. doi: 10.1016/j.ijrobp.2010.07.009 20932664

[4] Langmack KA Perry C Sinstead C Mills J Saunders D. The utility of atlas-assisted segmentation in the male pelvis is dependent on the interobserver agreement of the structures segmented. Br J Radiol (2014) 87:20140299. doi: 10.1259/bjr.20140299 25168198 PMC4207155

[5] Sharp G Fritscher KD Pekar V Peroni M Shusharina N Veeraraghavan H. Vision 20/20: Perspectives on automated image segmentation for radiotherapy. Med Phys (2014) 41:50902. doi: 10.1118/1.4871620 PMC4000389 24784366

[6] Sykes J. Reflections on the current status of commercial automated segmentation systems in clinical practice. J Med Radiat Sci (2014) 61:131–4. doi: 10.1002/jmrs.65 PMC4175848 26229648

[7] Hwee J Louie AV Gaede S Bauman G D'Souza D Sexton T. Technology assessment of automated atlas based segmentation in prostate bed contouring. Radiat Oncol (2011) 6:110. doi: 10.1186/1748-717X-6-110 21906279 PMC3180272

[8] Anders LC Stieler F Siebenlist K Schafer J Lohr F Wenz F. Performance of an atlas-based autosegmentation software for delineation of target volumes for radiotherapy of breast and anorectal cancer. Radiother Oncol (2012) 102:68–73. doi: 10.1016/j.radonc.2011.08.043 21962822

[9] Warfield SK Zou KH Wells WM. Simultaneous truth and performance level estimation (STAPLE): An algorithm for the validation of image segmentation. IEEE Trans Med Imaging (2004) 23:903–21. doi: 10.1109/TMI.2004.828354 PMC1283110 15250643

[10] La Macchia M Fellin F Amichetti M Cianchetti M Gianolini S Paola V. Systematic evaluation of three different commercial software solutions for automatic segmentation for adaptive therapy in head-and-neck, prostate and pleural cancer. Radiat Oncol (2012) 7:160. doi: 10.1186/1748-717X-7-160 22989046 PMC3493511

[11] Wong WK Leung LH Kwong DL. Evaluation and optimization of the parameters used in multiple-atlas-based segmentation of prostate cancers in radiation therapy. Br J Radiol (2016) 89:20140732. doi: 10.1259/bjr.20140732 26539630 PMC4985939

[12] Greenham S Dean J Fu CK Goman J Mulligan J Tune D. Evaluation of atlas-based auto-segmentation software in prostate cancer patients. J Med Radiat Sci (2014) 61:151–8. doi: 10.1002/jmrs.64 PMC4175851 26229651

[13] Sjoberg C Lundmark M Granberg C Johansson S Ahnesjo A Montelius A. Clinical evaluation of multi-atlas based segmentation of lymph node regions in head and neck and prostate cancer patients. Radiat Oncol (2013) 8:229. doi: 10.1186/1748-717X-8-229 24090107 PMC3842681

[14] Kim N Chang JS Kim YB Kim JS. Atlas-based auto-segmentation for postoperative radiotherapy planning in endometrial and cervical cancers. Radiat Oncol (2020) 15:106. doi: 10.1186/s13014-020-01562-y 32404123 PMC7218589

[15] Jhingran A Winter K Portelance L Miller B Salehpour M Gaur R. A phase II study of intensity modulated radiation therapy to the pelvis for postoperative patients with endometrial carcinoma: Radiation therapy oncology group trial 0418. Int J Radiat Oncol (2012) 84:E23–8. doi: 10.1016/j.ijrobp.2012.02.044 22543211

[16] Lim K Small WJ Portelance L Creutzberg C Jurgenliemk-Schulz IM Mundt A. Consensus guidelines for delineation of clinical target volume for intensity-modulated pelvic radiotherapy for the definitive treatment of cervix cancer. Int J Radiat Oncol Biol Phys (2011) 79:348–55. doi: 10.1016/j.ijrobp.2009.10.075 20472347

[17] Small WJ Bosch WR Harkenrider MM Strauss JB Abu-Rustum N Albuquerque KV. NRG Oncology/RTOG consensus guidelines for delineation of clinical target volume for intensity modulated pelvic radiation therapy in postoperative treatment of endometrial and cervical cancer: An update. Int J Radiat Oncol Biol Phys (2021) 109:413–24. doi: 10.1016/j.ijrobp.2020.08.061 PMC7856050 32905846

[18] Lee H Lee E Kim N Kim JH Park K Lee H. Clinical evaluation of commercial atlas-based auto-segmentation in the head and neck region. Front Oncol (2019) 9:239. doi: 10.3389/fonc.2019.00239 31024843 PMC6465886

[19] Voet PW Dirkx ML Teguh DN Hoogeman MS Levendag PC Heijmen BJ. Does atlas-based autosegmentation of neck levels require subsequent manual contour editing to avoid risk of severe target underdosage? a dosimetric analysis. Radiother Oncol (2011) 98:373–7. doi: 10.1016/j.radonc.2010.11.017 21269714

[20] Casati M Piffer S Calusi S Marrazzo L Simontacchi G Di Cataldo V. Methodological approach to create an atlas using a commercial auto-contouring software. J Appl Clin Med Phys (2020) 21:219–30. doi: 10.1002/acm2.13093 PMC7769405 33236827

[21] Casati M Piffer S Calusi S Marrazzo L Simontacchi G Di Cataldo V. Clinical validation of an automatic atlas-based segmentation tool for male pelvis CT images. J Appl Clin Med Phys (2022) 23:e13507. doi: 10.1002/acm2.13507 35064746 PMC8906199

[22] Ducote JL Sehgal V Wong J Al-Ghazi M. SU-E-J-102: The impact of the number of subjects for atlas-based automatic segmentation. Med Phys (2012) 39:3676. doi: 10.1118/1.4734938 28519803

[23] Luddemann T Egger J. Iterative-cuts: Longitudinal and scale-invariant segmentation *via* user-defined templates for rectosigmoid colon in gynecological brachytherapy. J Med Imaging (Bellingham) (2016) 3:24004. doi: 10.1117/1.JMI.3.2.024004 PMC4912606 27403448

[24] Lin L Dou Q Jin YM Zhou GQ Tang YQ Chen WL. Deep learning for automated contouring of primary tumor volumes by MRI for nasopharyngeal carcinoma. Radiology (2019) 291:677–86. doi: 10.1148/radiol.2019182012 30912722

[25] Kim JR Shim WH Yoon HM Hong SH Lee JS Cho YA. Computerized bone age estimation using deep learning based program: Evaluation of the accuracy and efficiency. AJR Am J Roentgenol (2017) 209:1374–80. doi: 10.2214/AJR.17.18224 28898126

[26] Nam JG Park S Hwang EJ Lee JH Jin KN Lim KY. Development and validation of deep learning-based automatic detection algorithm for malignant pulmonary nodules on chest radiographs. Radiology (2019) 290:218–28. doi: 10.1148/radiol.2018180237 30251934

[27] Steiner DF MacDonald R Liu Y Truszkowski P Hipp JD Gammage C. Impact of deep learning assistance on the histopathologic review of lymph nodes for metastatic breast cancer. Am J Surg Pathol (2018) 42:1636–46. doi: 10.1097/PAS.0000000000001151 PMC6257102 30312179

[28] Ding Y Chen Z Wang Z Wang X Hu D Ma P. Three-dimensional deep neural network for automatic delineation of cervical cancer in planning computed tomography images. J Appl Clin Med Phys (2022) 23:e13566. doi: 10.1002/acm2.13566 35192243 PMC8992957

[29] Yoganathan SA Paul SN Paloor S Torfeh T Chandramouli SH Hammoud R. Automatic segmentation of magnetic resonance images for high-dose-rate cervical cancer brachytherapy using deep learning. Med Phys (2022) 49:1571–84. doi: 10.1002/mp.15506 35094405

[30] Liu Z Liu X Xiao B Wang S Miao Z Sun Y. Segmentation of organs-at-risk in cervical cancer CT images with a convolutional neural network. Phys Med (2020) 69:184–91. doi: 10.1016/j.ejmp.2019.12.008 31918371

